# PReS-FINAL-2103: Real life management of VZV infection or reactivation in rheumatic children on biologics: the French experience

**DOI:** 10.1186/1546-0096-11-S2-P115

**Published:** 2013-12-05

**Authors:** S Guillaume-Czitrom, V Remy-Piccolo, A Duquesne, I Kone-Paut, M Grall, E Allain-Launay, R Dagher, A Faye, K Brochard, E Grouteau, C Pietrement, L Rossi, P Quartier, J Morel

**Affiliations:** 1 Pediatric Rheumatology, Bicetre Hospital, Kremlin Bicetre, Paris, France; 2 Pediatric Rheumatology, Necker Hospital, Paris, France; 3 Pediatric Rheumatology, HFME, Lyon, France; 4 Pediatrics, Rouen Hospital, Rouen, France; 5 Pediatric Nephrology, Nantes Hospital, Nantes, France; 6 Pediatrics, Byblos Hospital, Byblos, Lebanon; 7 Pediatrics, Robert Debre Hospital, Paris, France; 8 Pediatric Nephrology, Toulouse Hospital, Toulouse, France; 9 Pediatrics, Toulouse Hospital, Toulouse, France; 10 Pediatrics, Reims Hospital, Reims, France; 11 Rheumatology, Montpellier Hospital, Montpellier, France

## Introduction

Treatments of severe rheumatic conditions in children are increasingly based on biologics. The most common adverse events on all kind of biologics are mild infections in ENT, pulmonary and urinary systems. The number of cases of varicella-zoster-virus (VZV) manifestations while being on biologics is constantly growing. No consensus exists about what to do when facing this situation yet.

## Objectives

The aim of this work is to describe the French experience of VZV infection/reactivation management in children affected with chronic inflammatory rheumatisms and treated with available biologics in a real life setting.

## Methods

French pediatric rheumatologists were asked through the web site of the Société Francophone des rhumatismes et maladies inflammatoires de l'enfant -SOFREMIP- by phone and/or e-mail to report their cases of VZV manifestations on biologics using a standardized and anonymized e-CRF.

## Results

21 cases of VZV infection/reactivation were reported in children less than 18 yrs, followed for rheumatic conditions treated with biotherapies. 19 had JIAs of which 9 had systemic JIAs, 1 had SLE, 1 had polyarthritis with immune deficiency. Biologics-associated treatments were NSAIDs in 5 cases, steroids in 9 cases, DMARDs in 14 (MTX in 12, MMF and HCQ in 1, AZA in 1). Biologics were anti-TNFs in 13 children (9 ETN, 4 ADA), anti-IL1 in 6 (4 CNK, 2 ANK), 1 had TCZ, and 1 RTX. A long delay was observed between the initiation of biologics and the VZV manifestations in the majority of patients, whatever the biologic. None of our cases was vaccinated against VZV. 12 children had varicella (7 with concurrent anti-TNF treatment, 4 with anti-IL1, 1 with RTX) and 9 patients had herpes zoster (6 with anti-TNFs, 2 with anti-IL1, 1 with TCZ). 2 patients had a complicated clinical picture of VZV infection/reactivation: 1 had an ophthalmic zoster and 1 had a systemic dissemination of VZV with severe hepatic involvement and pancytopenia. NSAIDs were stopped 4 times out of 5 continuous treatment, steroids were stopped in 1/9 patients, DMARDs were interrupted in 8/14, and biologics in 15/21 cases. 16/21 patients received a specific anti-herpetic drug despite a common non-complicated outcome, whereas 5 children were not treated in the same situation (3 on anti-TNFs, 2 on anti-IL1). All patients had favorable outcomes.

## Conclusion

This study shows an overrepresentation of systemic JIAs among patients infected by or reactivating the VZV. This group of patients may be more immunosuppressed than others. In spite of the great heterogeneity of VZV management in France in children treated with biologics, the outcome was invariably good. This suggests 2 important points (i) VZV control may not need any of the cytokines/pathways targeted by the biologics we use, (ii) VZV management on biologics should be rationalized.

## Disclosure of interest

None declared.

